# An Intriguing Case of Myocardial Deposits

**DOI:** 10.7759/cureus.67226

**Published:** 2024-08-19

**Authors:** Arpita Pawa, Saloni Savani, Michael Chammany, Saahir Mukherjee, Kathryn Gayle

**Affiliations:** 1 Internal Medicine, Willis Knighton Health, Shreveport, USA; 2 Medical School, Edward Via College of Osteopathic Medicine, Monroe, USA; 3 Advanced Cardiac Imaging, Willis Knighton Health, Shreveport, USA

**Keywords:** choledocholithiasis, cholangiocarcinoma, myocardial fibrosis, mri cardiac, dystrophic myocardial calcification

## Abstract

Cardiac calcification refers to calcium deposits in the coronary arteries, heart valves, pericardium, or myocardium. Calcium deposition within the myocardium is unique and can be secondary to metastatic or dystrophic calcification. Both forms are linked to cardiac abnormalities and poor prognosis. The most common causes include myocardial infarction, sepsis, myocarditis, renal failure, and hyperparathyroidism. Here, we report the case of a 74-year-old male who was found to have gallbladder adenocarcinoma with subsequent preoperative workup indicating possible metastases to the myocardium. With the use of multimodality imaging, particularly cardiac MRI, the differentiation between metastatic disease and intramyocardial calcification was made. The case aims to highlight the complexity of diagnosing and managing myocardial calcifications and underscores the need for further research into their etiology and implications.

## Introduction

Myocardial calcification is generally a rare finding in imaging, and the etiology of such a process can be elucidated by a wide variety of normal physiological or pathological mechanisms. Calcification can occur either dystrophically, usually due to some disturbance in calcium homeostasis such as local tissue damage, or metastatically, where movement of calcium salts is altered, leading to hypercalcemia and subsequent deposition in tissue [1]. Myocardial calcification has been associated with cardiac abnormalities, like arrhythmias, and alterations in normal cardiac anatomy, such as chamber dilation and valvular dysfunctions [2]. Therefore, long-term prognosis is often poor and associated with high mortality [2].

Dystrophic calcification accounts for the majority of cardiac calcification that is detected on imaging. This type of calcification represents the sequelae of local tissue damage [3]. Therefore, dystrophic calcification is often seen in the context of ventricular aneurysms after myocardial infarction, with calcium often accumulating in the infarcted tissue during the myocardial healing process [1]. It can also occur as a complication of cardiac surgery [1]. Pathological dystrophic calcification is extremely complex, but it is thought to arise due to soft tissue alkalinity that binds ionic calcium [1]. Dystrophic calcification only occurs in necrotic areas [4].

Metastatic myocardial calcification, on the other hand, is more common and is associated with pathological conditions that are associated with hypercalcemia; chronic kidney disease or renal failure, hyperparathyroidism, sepsis, and malignancies are such examples [4]. It can also occur with processes that are associated with resorption of bone, such as osteomyelitis or metastatic tumors [1]. The underlying pathophysiology may be associated with initial mitochondrial calcification of muscle fibers, in which calcium homeostasis is disrupted from disturbance in normal mitochondrial function [1]. Dystrophic or metastatic myocardial calcification is associated with a variety of factors and processes, and while the process in itself may be debilitating due to its association with cardiac dysfunction, its presence may be indicative of an underlying or more insidious process.

## Case presentation

A 74-year-old man presented to the hospital with discomfort in his right upper abdomen, nausea, and vomiting. His medical history was significant for asthma, hypertension, uncomplicated type 2 diabetes mellitus, and stage 3 chronic kidney disease. His family had a significant cancer history, with his sister and mother both having breast cancer and his father having lung cancer. Additionally, he reported an unintended weight loss of 25 pounds over two months. Laboratory tests revealed elevated liver enzymes and a magnetic resonance cholangiopancreatography (MRCP) identified a stone lodged within the patient’s distal common bile duct. Endoscopic retrograde cholangiography (ERCP) led to the placement of a stent within the common bile duct, followed by an open cholecystectomy the next day. Pathological examination of the gallbladder showed moderately differentiated adenocarcinoma invading through the muscular layer into the perimuscular connective tissue on the hepatic side without liver involvement. The cystic duct margin was positive for invasive carcinoma. Hematology-Oncology was consulted and suggested further staging with a positron-emission-therapy (PET) scan and definitive treatment with gallbladder bed resection and lymph node removal. Additional screening was ordered, including next-generation sequencing and cancer antigen 19-9 (CA19-9). The patient was referred to hepatobiliary surgery, and the appropriate screenings were ordered to prepare the patient for surgery.

**Figure 1:**
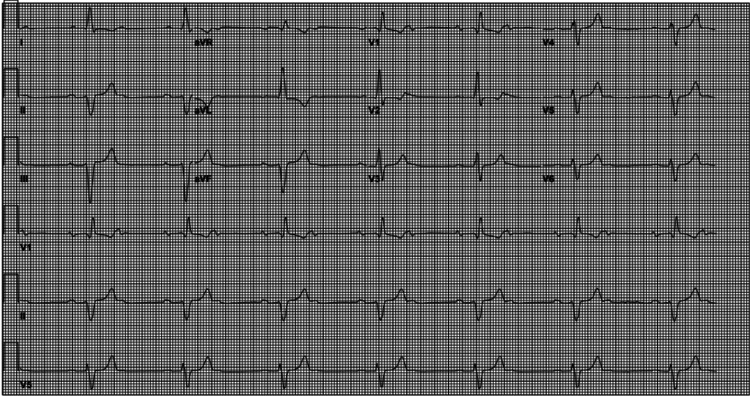
ECG showing sinus bradycardia with second degree A-V Block with 2:1 A-V conduction, left axis deviation, and right bundle branch block

During preoperative workup, an electrocardiogram revealed sinus bradycardia with 2:1 atrioventricular conduction block, along with left axis deviation and right branch bundle block as shown in Figure 1. Transthoracic echocardiogram demonstrated moderate concentric left ventricular hypertrophy, anterior and apical left ventricle (LV) wall motion hypokinesis, mildly enlarged left and right atria, and fibrocalcific changes, and mild regurgitation noted across the aortic, mitral, and tricuspid valves (see Table 1 in the Appendix for further details). A nuclear medicine stress test revealed a depressed left ventricular ejection fraction (LVEF) of 38% with possible mild ischemic changes in the distal anterior and inferior walls. The patient was asymptomatic and hemodynamically stable despite these abnormal findings.

**Figure 2:**
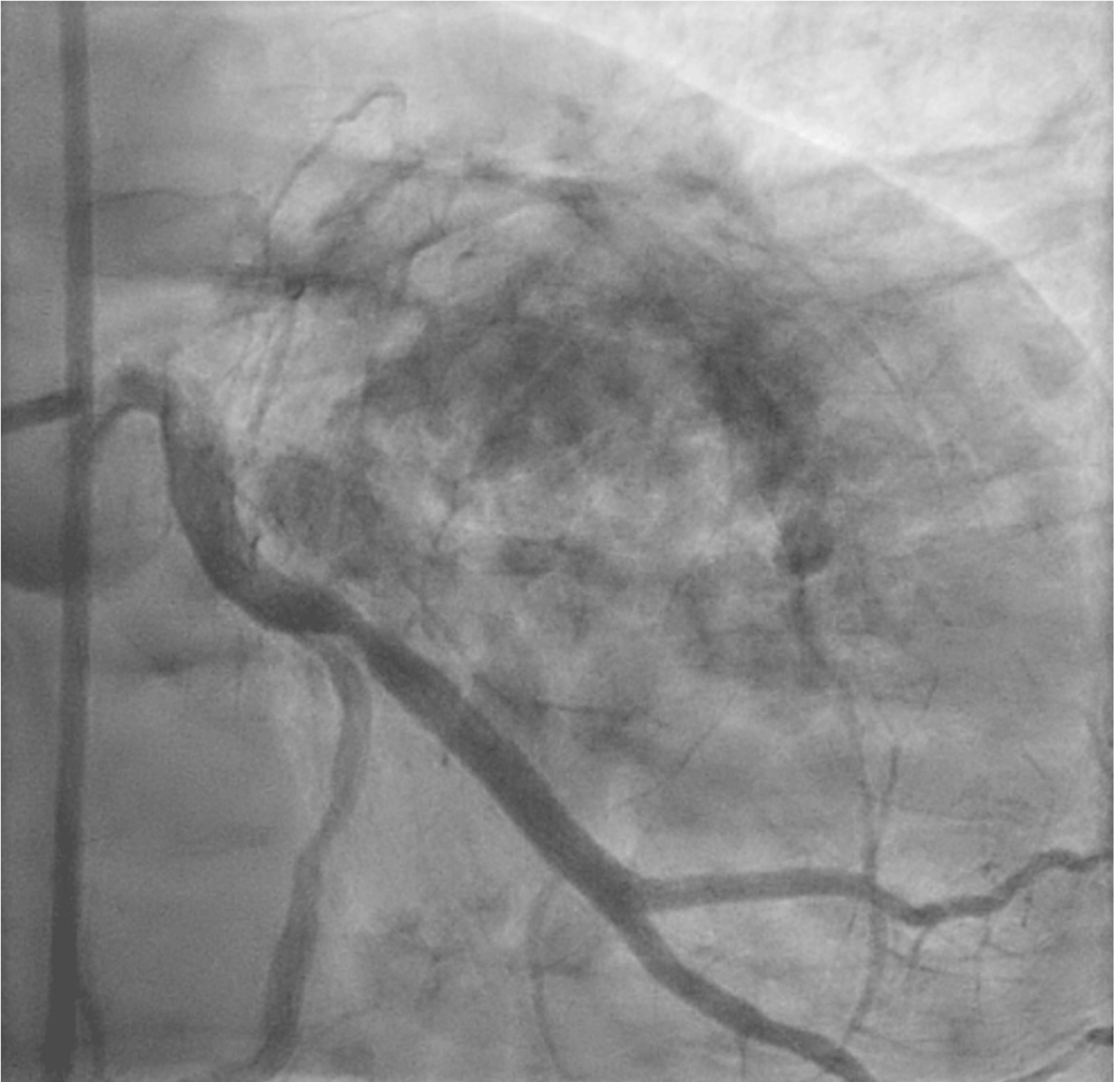
Contrast-induced enhancement in the LV myocardium as seen on left heart catheterization LV: Left ventricle

**Figure 3:**
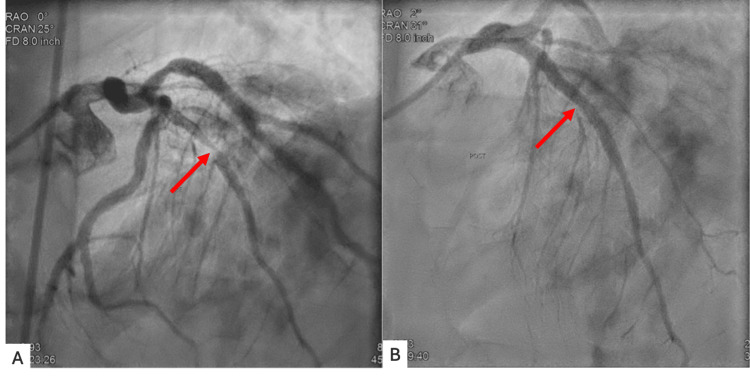
Figure 3A shows the occluded LAD prior to stenting. Figure 3B shows patent LAD after stenting LAD: Left anterior descending artery

**Figure 4:**
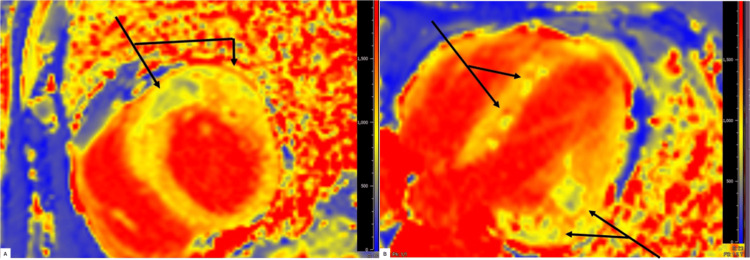
(A and B) Cardiac MRI showing very low T1 time in areas of calcium deposition. T1 here is 673 msec, whereas normal T1 is between 940 and 1030 msec

**Figure 5:**
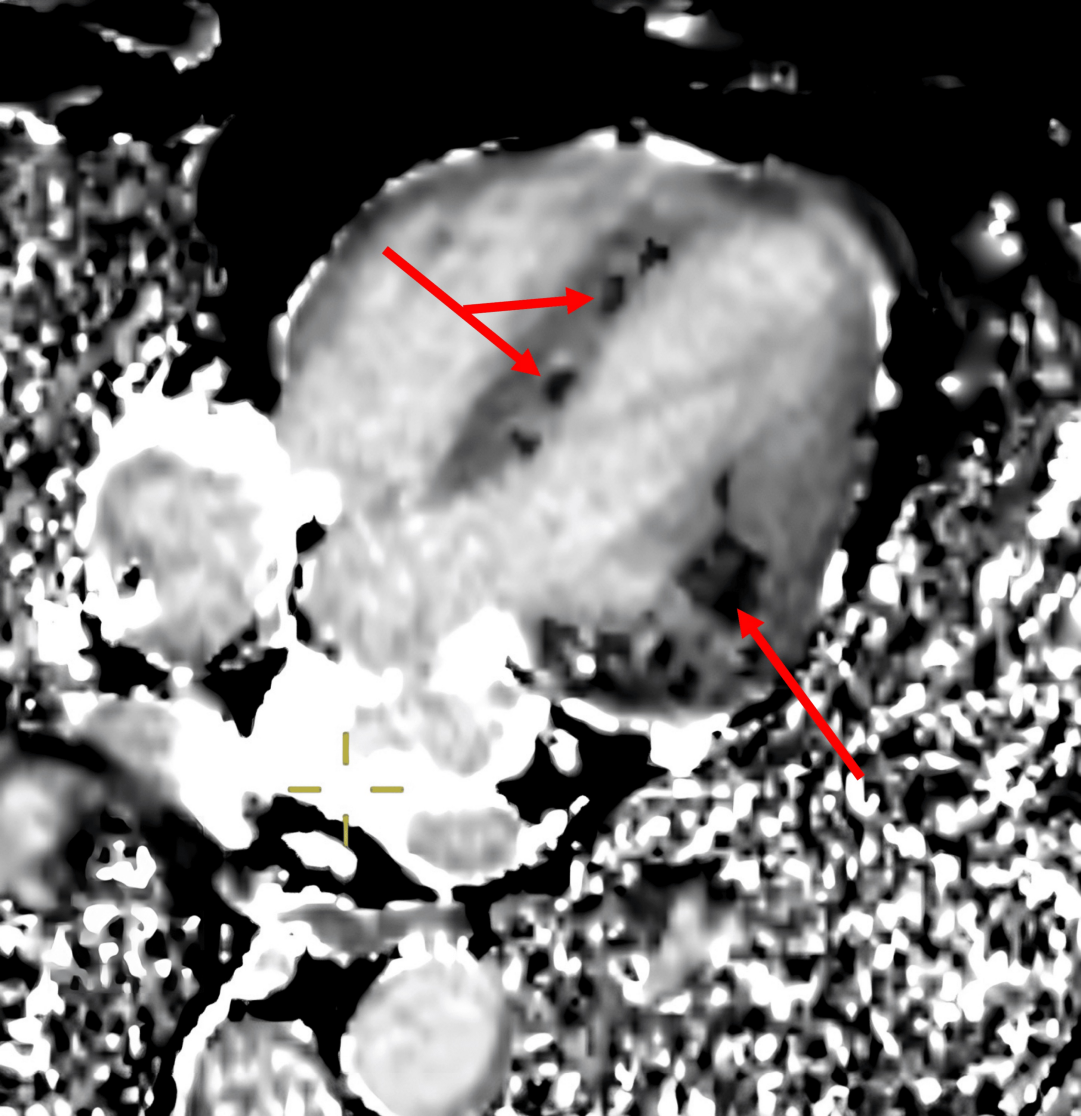
Grey scale T1 image showing calcium as black deposits

Given a positive stress test, subsequent cardiac catheterization with ventriculography and coronary angiography revealed peculiar findings as shown in Figure 2. On ventriculography, the patient’s LV looked bizarre, with contrast-induced enhancements indicating possible cancer involvement. Coronary angiography revealed the left main coronary artery to be calcified but functioning normally, and the left anterior descending (LAD) artery was 95% occluded, marking it as a critical lesion. Following catheterization and stent placement in the LAD as shown in Figure 3, the patient was started on rosuvastatin 20 mg and dual antiplatelet therapy with aspirin 81 mg daily and Brilinta 90 mg twice a day. To further evaluate the nature of the myocardial deposits, cardiac magnetic resonance imaging (MRI) was performed which revealed a mildly enlarged LV with hyperdynamic systolic function; LVEF was noted to be 78%. Multiple intramyocardial masses were noted with low T1 and low T2 signals and no late gadolinium enhancement, indicating the presence of intramyocardial calcifications rather than metastatic disease (Figures 4-5). Myocardial fibrosis and scarring were noted surrounding the intramyocardial nodules, though there was no evidence of infiltration or inflammation. Aside from abnormalities noted in the heart, a hepatic mass and dense hilar lymphadenopathy were revealed as well. 

The patient underwent percutaneous coronary intervention (PCI) with successful stent placement in the LAD, followed by permanent pacemaker implantation to address his persistent bradycardia. The pacemaker placement occurred successfully with no complications, and the patient remained hemodynamically stable, except for elevated blood pressure. To address this, he was started on hydralazine 25 mg twice a day and nifedipine 30 mg once daily along with the losartan 12.5 mg once daily that he had already been receiving prior to admission. The patient was advised to continue his dual antiplatelet therapy regimen for an additional month, after which he would be reassessed by the surgical team to determine his eligibility for hepatobiliary surgery. Two months later, he underwent hepatobiliary surgery, which included gallbladder fossa resection, partial hepatectomy, omental resection, re-excision of the cystic duct with clear margins, biliary stent placement, and lymphadenectomy. Postoperative PET/computed tomography (CT) (Figures 3-4) revealed no evidence of distant metastatic disease, but extensive myocardial calcifications remained. He was discharged shortly after surgery without any complications.

**Figure 6:**
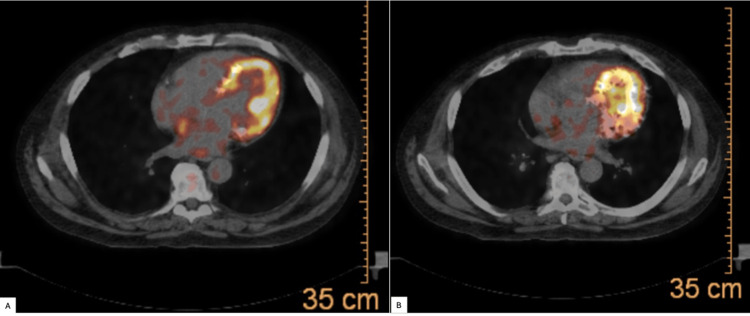
(A and B) Positron emission therapy (PET) scan showing a metabolically active myocardium which is normal; however, abnormal areas of calcification can be seen within this normally metabolically active heart

**Figure 7:**
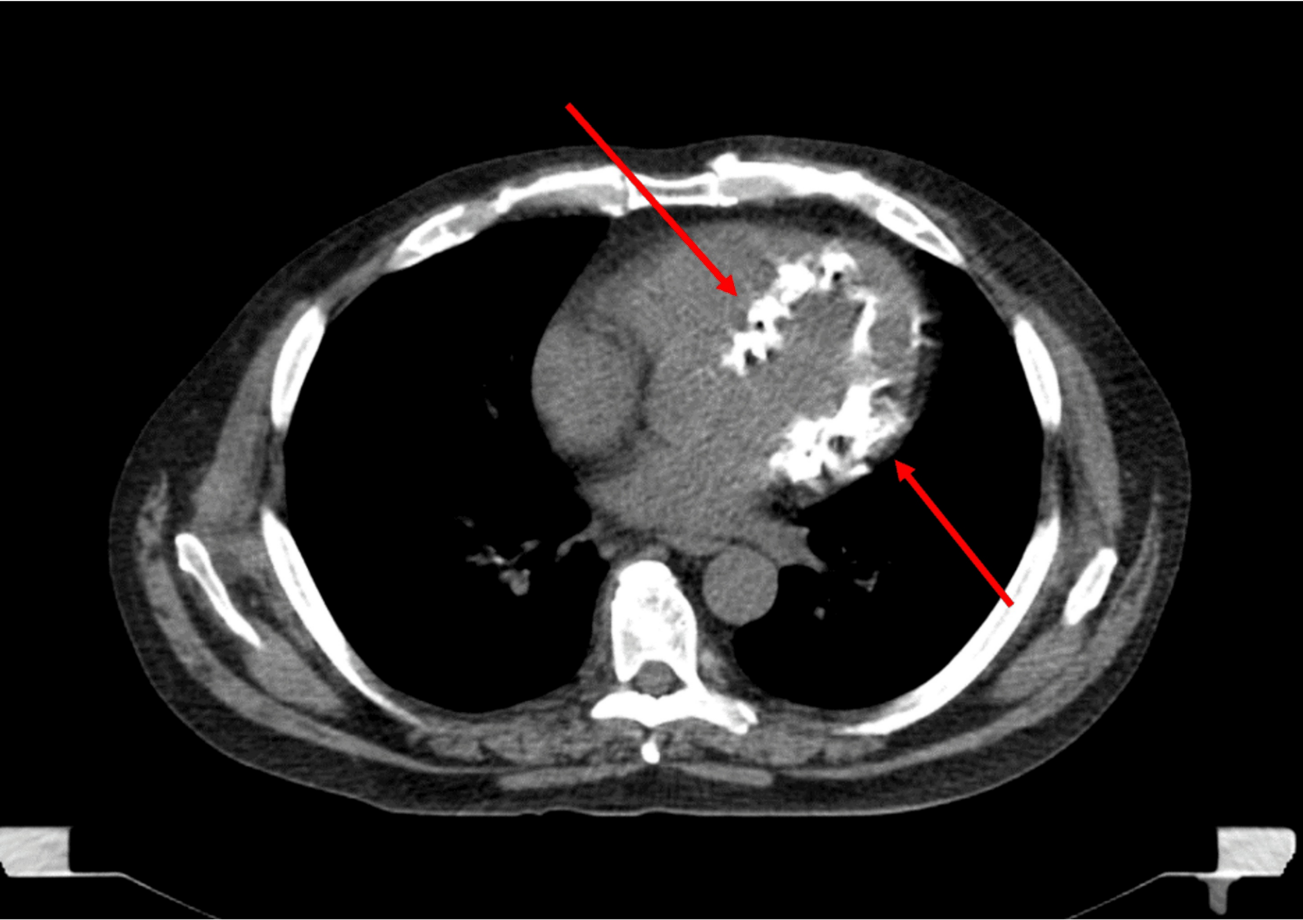
Low-dose CT scan showing bright calcium deposits within the myocardium. Calcium appears as bright as bone CT: Computed tomography

## Discussion

Our case illustrates the insidious yet extensive nature of intramyocardial calcifications. Despite our patient exhibiting a myriad of abnormalities associated with the architecture of his heart, they were only discovered incidentally during hepatobiliary surgery preoperative screening, manifesting as persistent but asymptomatic bradycardia and 2:1 atrioventricular (AV) block. With complete resection of the patient’s gallbladder, stent placement within the LAD, and placement of a permanent pacemaker, the patient’s complications were well managed, despite extensive calcifications remaining within the patient’s LV. Questions persist regarding the calcification’s etiology, particularly whether they originated from dystrophic or metastatic pathways. The lack of elevated T2 signal and poor uptake of late gadolinium enhancement (LGE) during cardiac MRI prior to hepatobiliary surgery ruled out metastatic myocardial calcification [5]. Furthermore, the patient's serum calcium levels were generally normal, strengthening the notion that the cause of the calcification was not due to metastatic involvement from cholangiocarcinoma [6].

Further exploration into dystrophic intramyocardial calcification etiologies is warranted due to its sinister presentation. Most reported cases of myocardial calcifications are identified postmortem and are primarily associated with severe coronary artery disease and myocardial infarction in elderly men [7]. Furthermore, global cardiac injuries stemming from etiologies such as ischemia, sepsis, chronic renal failure, and infections (e.g., tuberculosis) can result in intramyocardial calcification formation [8,9]. Despite the absence of severe coronary artery injury, our patient’s past medical history was notable for hypertension and type 2 diabetes mellitus, and further studies should be conducted to investigate the role of these pathologies in the development of myocardial calcifications. Additionally, reports of dystrophic myocardial calcifications are commonly attributed to chronic kidney disease due to hyperphosphatemia, secondary hyperparathyroidism, and ultimately, calcium deposition within the walls of the heart [2,6,10].Although our patient had stage 3 chronic kidney disease, calcium and phosphorus concentrations were within reference ranges. Glomerular filtration rate remained just below 60, and creatinine measurements were elevated though relatively stable.

In general, the location of myocardial calcifications significantly influences their associated comorbidities and complications, leading to issues such as impaired diastolic filling, focal wall motion abnormalities, arrhythmias, and myocardial dysfunction [9,10]. Treatment and prognosis remain poorly defined in literature, underscoring the need for further research to elucidate the etiologies, morbidities, and prognosis associated with extensive myocardial calcifications and to guide more effective management strategies.

## Conclusions

Myocardial calcium deposition is not a well-understood process and warrants further research and evaluation. The mechanisms associated with this condition are extensive, and multiple etiologies can contribute to calcium deposits in soft tissue. Although calcium deposition in the myocardium may be associated with impaired cardiac function, it remains an important prognostic factor and may be indicative of a more serious underlying pathology. Differentiating between metastatic and dystrophic myocardial calcification is complex, and these pathophysiological processes may overlap, especially in this patient who presented to the hospital with a complex past medical and surgical history. 

## Appendices

**Table 1:** Transthoracic echocardiography

Indication	Bradycardia
Left ventricle (LV)	Left ventricular cavity size is normal. There is moderate left ventricular hypertrophy. Global systolic function: normal systolic function; left ventricular EF 55%-60%. Regional systolic function: anterior LV wall motion is hypokinetic, apical LV wall motion is hypokinetic
Right ventricle	Normal right ventricular size and systolic function
Left atrium	The left atrial size is mildly increased
Right atrium	The right atrium is mildly enlarged
Aortic valve	Fibrocalcific changes of the aortic valve. There is no evidence of aortic stenosis. There is mild aortic regurgitation
Mitral valve (MV)	Fibrocalcific changes of the MV. Mild mitral annular calcification present. There is no mitral stenosis. There is mitral regurgitation
Tricuspid valve	Mild to moderate tricuspid regurgitation present. The RSVP is estimated at 41.5 mmHg, which is mildly elevated
Aorta/aortic root	The aortic root is normal in size with atherosclerosis
IVC/Hepatic Veins	The inferior vena cava is dilated with >50% inspiratory collapse
Pericardium	There is no pericardial effusion
